# Resolution of Bullous Pemphigoid Following Lung Cancer Resection: A Case of Paraneoplastic Pemphigoid

**DOI:** 10.7759/cureus.73485

**Published:** 2024-11-11

**Authors:** Joohyung Youh, Takuya Mizukami, Yuri Nagata, Kei Ito

**Affiliations:** 1 Department of Dermatology, JR Sapporo Hospital, Sapporo, JPN

**Keywords:** anti bp180 antibody, bullous pemphigoid, lung cancer, paraneoplastic pemphigoid, squamous cell carcinoma of lung

## Abstract

Bullous pemphigoid (BP) is a chronic autoimmune disorder characterized by subepidermal blister formation, primarily affecting elderly individuals. While BP has been associated with malignancies, the exact nature of this relationship remains unclear. We report the case of a 72-year-old man who presented with pruritic cutaneous lesions, including tense vesicles and bullae and was diagnosed with BP. Despite treatment with doxycycline, nicotinamide, and topical clobetasol, his symptoms persisted. A routine chest X-ray, conducted as part of his diagnostic workup, incidentally, revealed a large mass in the right upper lung, which was subsequently diagnosed as squamous cell carcinoma. Remarkably, after the surgical resection of the tumor, the patient’s BP lesions completely resolved within seven weeks, without alterations to his dermatologic treatment. This case emphasizes the importance of malignancy screening in patients with persistent BP and suggests a possible link between BP and underlying cancer, particularly when standard therapies prove ineffective. Further investigation into the mechanisms connecting these conditions is warranted.

## Introduction

Bullous pemphigoid (BP) is the most common autoimmune subepidermal blistering disorder, predominantly affecting older adults and typically presenting as generalized pruritic bullous eruptions. The condition can lead to significant morbidity. Clinical presentations of BP can vary, especially in the early stages or in atypical cases where bullae may not fully form. BP is driven by an immune response targeting two key self-antigens: BP180 and BP230 [1].

Lucariello et al. reported an 11% incidence of malignancy in patients with BP, suggesting a notable coexistence of BP and malignancies [2]. Some cases of paraneoplastic BP are refractory to standard therapies, including corticosteroids and immunosuppressants. However, there have been reports of rapid BP symptom resolution following the treatment of the underlying malignancy, such as lung cancer [3].

## Case presentation

**Figure 1:**
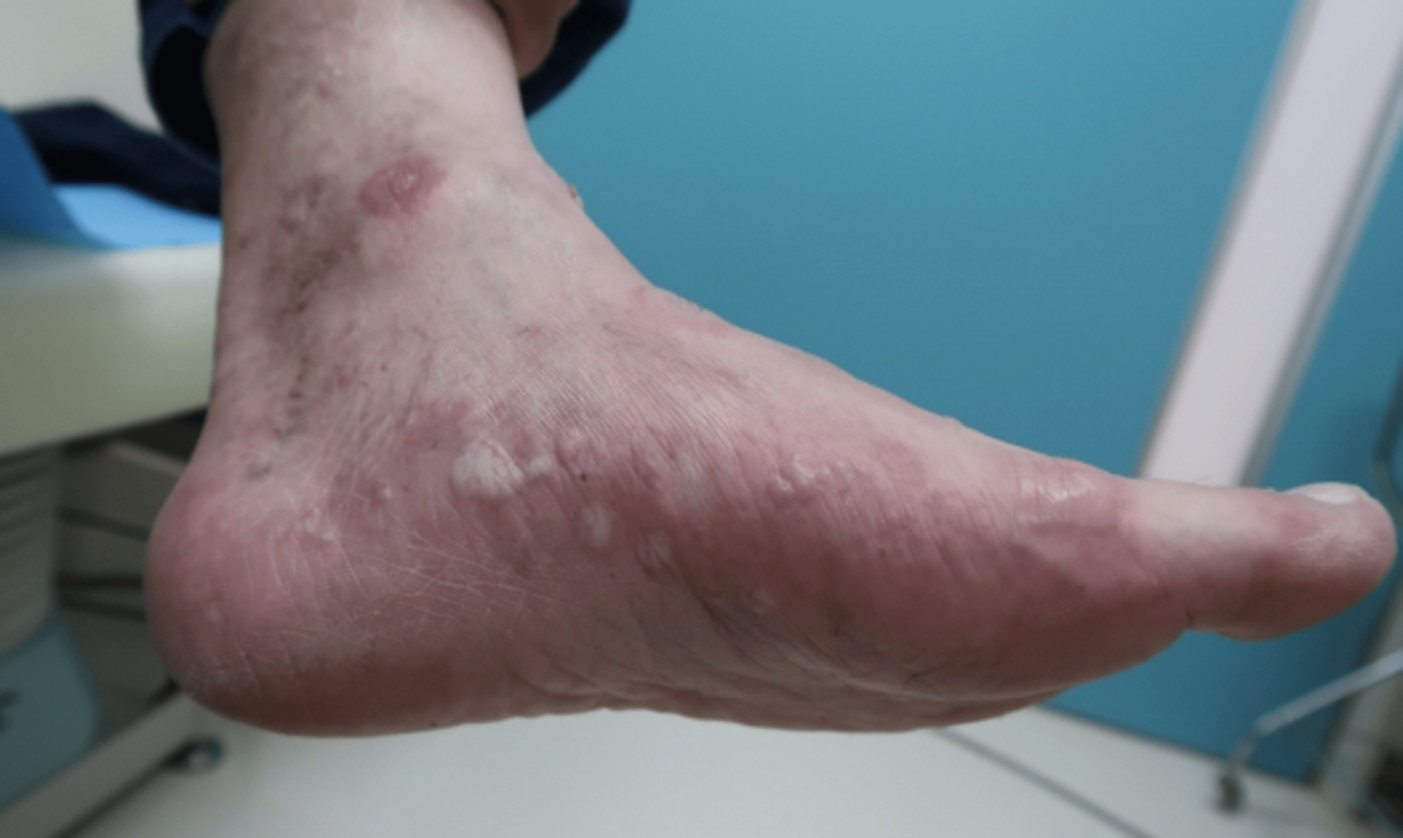
Initial presentation showing edematous erythema with tense blisters and pruritus, primarily affecting the extremities

A 72-year-old man with no significant medical history presented with pruritic cutaneous lesions and blisters on his trunk and extremities. Physical examination revealed erythematous urticarial plaques as well as tense vesicles and bullae of varying sizes filled with serous fluid (Figure 1). The Bullous Pemphigoid Disease Area Index (BPDAI) score at the initial visit was 37.

**Figure 2:**
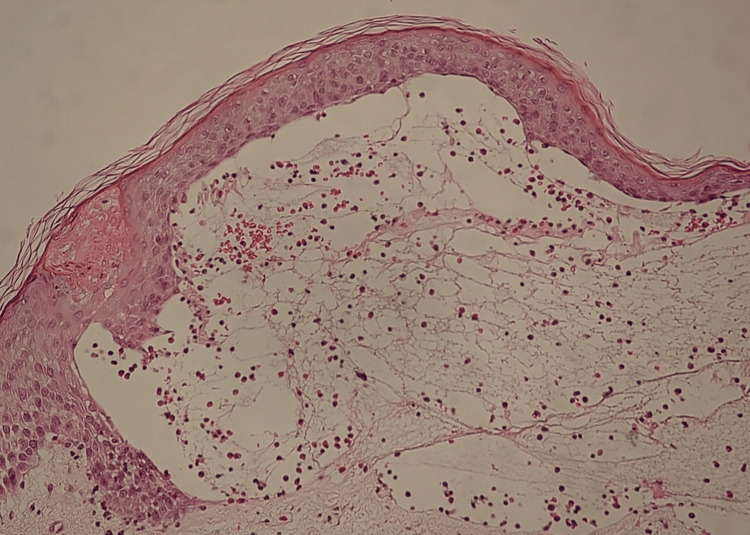
Histopathologic findings showing significant subepidermal blistering with eosinophilic infiltration (hematoxylin and eosin, 200x magnification)

A skin biopsy from a tense blister on an upper extremity demonstrated subepidermal blistering with eosinophilic infiltration (Figure 2).

**Figure 3:**
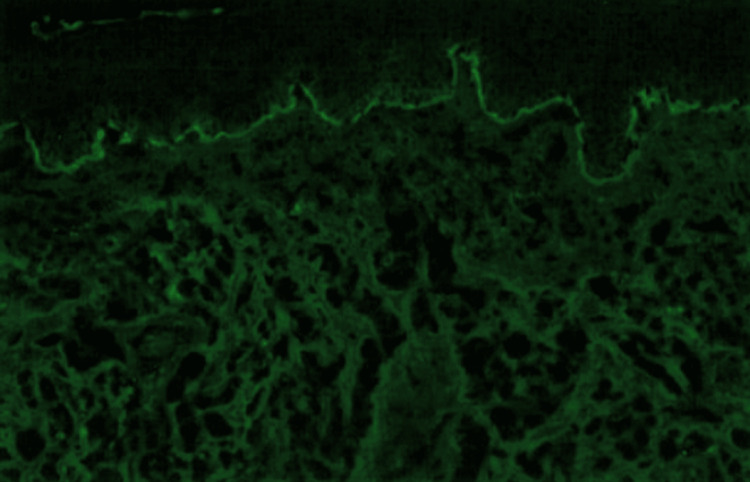
Direct immunofluorescence (DIF) showing linear deposition of C3 at the basement membrane zone

Direct immunofluorescence (DIF) revealed IgA (−), IgG (−), IgM (−), and C3 (+) with linear deposition along the basement membrane (Figure3).

The initial treatment consisted of doxycycline (200 mg/day), nicotinamide (900 mg/day), and topical clobetasol propionate (30 g daily). Despite this regimen, new blisters continued to form. As a result, we planned to initiate prednisolone at 0.5 mg/kg/day (30 mg/day) upon admission.

**Figure 4:**
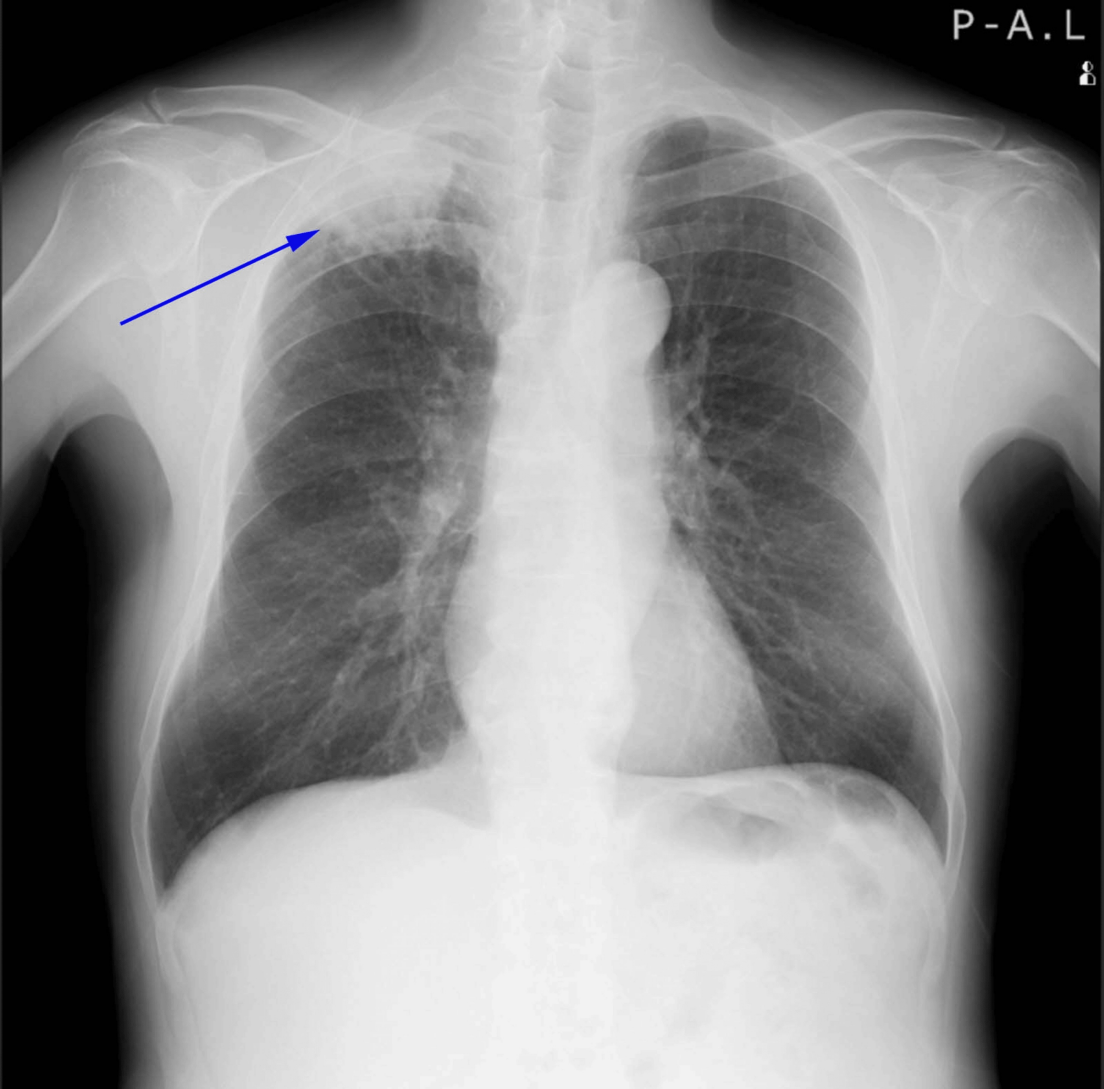
Routine chest X-ray on the day of admission showing a large mass (blue arrow) in the right upper lung

**Figure 5:**
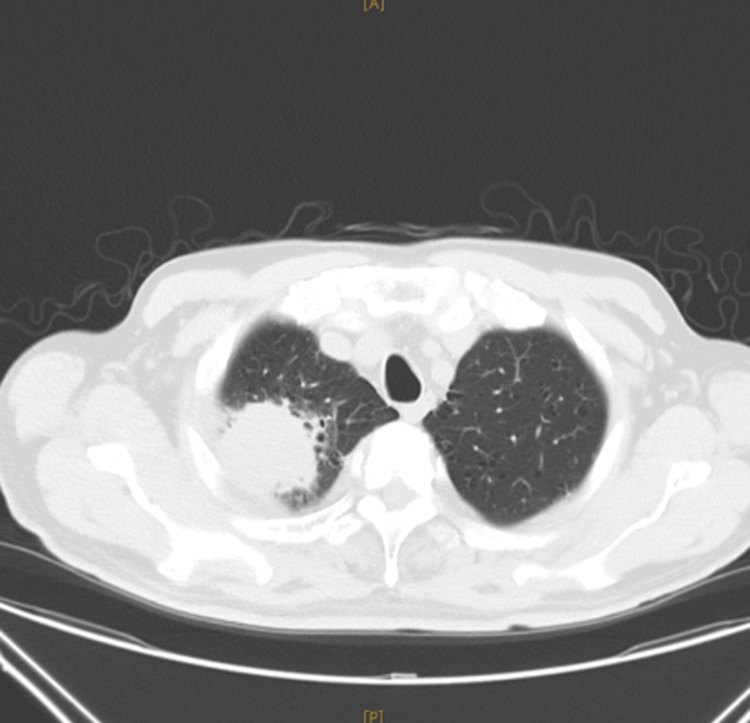
Computed tomography (CT) before resection of lung cancer showing a large mass in the right upper lung

As part of the diagnostic workup, a routine chest x-ray and electrocardiogram (EKG) were performed. The EKG showed no significant abnormalities, but the chest x-ray revealed a space-occupying lesion in the right upper lung (Figure 4). A subsequent chest computed tomography (CT) scan confirmed the presence of a large mass in the right upper lung (Figure 5).

A pulmonology consultation was arranged, and a bronchoscopic biopsy was performed. Histopathological and imaging findings confirmed the diagnosis of squamous cell carcinoma (SCC) of the lung (cT2bN2M0, Stage IIIA). The pulmonologist advised discontinuing the prednisolone to avoid interference with PET-CT results and reduce perioperative complications. The patient continued treatment for BP with doxycycline (200 mg/day), nicotinamide (900 mg/day), and topical clobetasol propionate (30 g daily).

**Figure 6:**
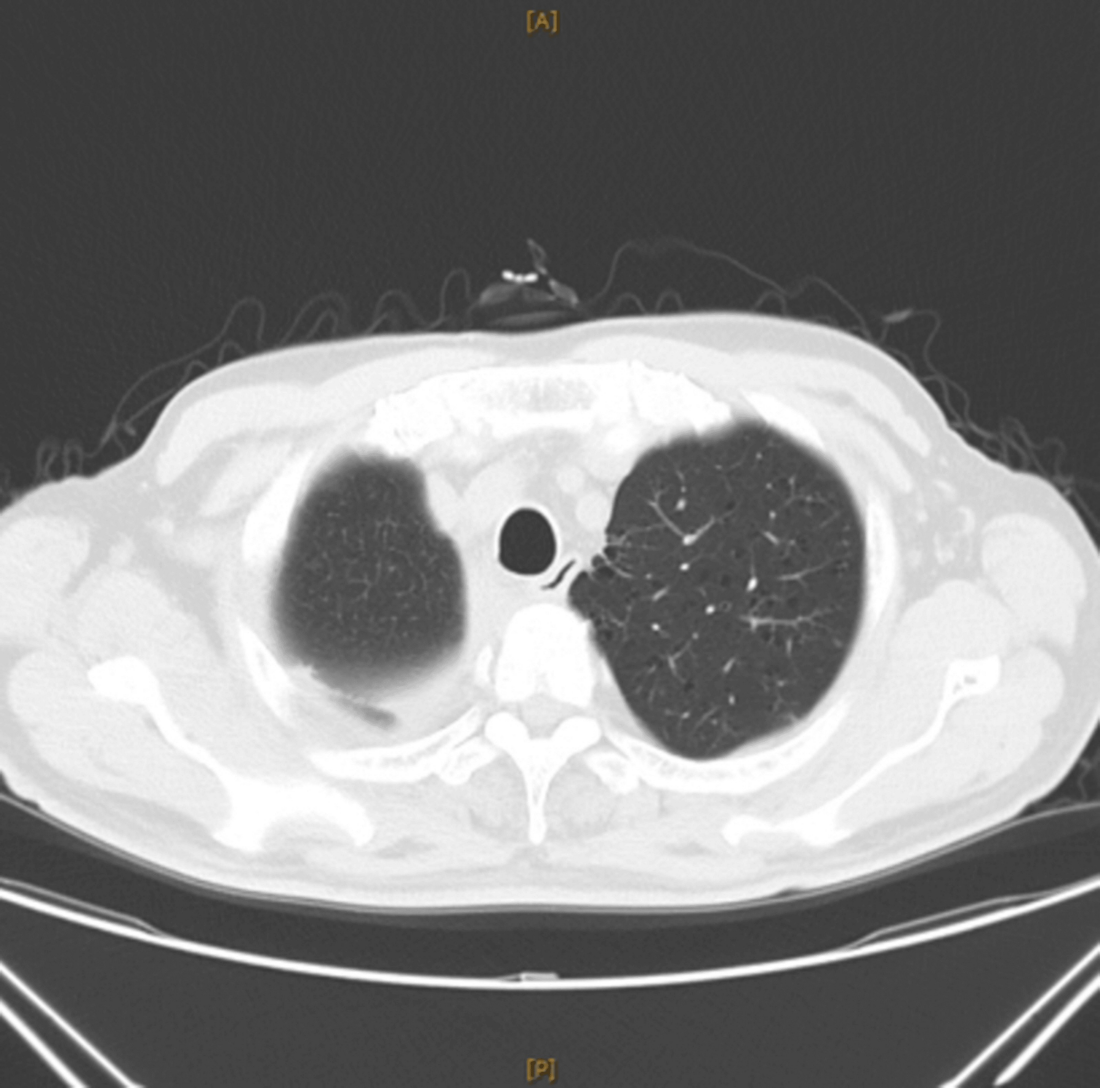
CT after resection of lung cancer showing no significant mass is observed in the right upper lung after tumor resection

Despite this treatment, there was no significant improvement in the cutaneous lesions, and the anti-BP180 antibody titer progressively increased. However, given the more immediate threat posed by the lung cancer, the regimen was maintained until surgical resection of the lung cancer. The cancer was completely resected (Figure 6), and since the patient declined adjuvant chemotherapy, no chemotherapy was administered.

At the one-month follow-up after tumor resection, laboratory tests - including the anti-BP180 antibody titer and BPDAI score - showed marked improvement (Table 1).

**Table 1:** Changes in anti-BP180 antibody titer and BPDAI score following lung cancer resection BPDAI: Bullous Pemphigoid Disease Area Index

Time point	Anti-BP180 antibody titer (U/mL)	BPDAI Score
Initial visit	390.3	37
3 weeks after initial visit	2170.0	27
5 weeks after initial visit	2320.0	25
3 weeks after tumor resection (9 weeks after initial visit)	191.8	5
7 weeks after tumor resection (13 weeks after initial visit)	63.5	0

**Figure 7:**
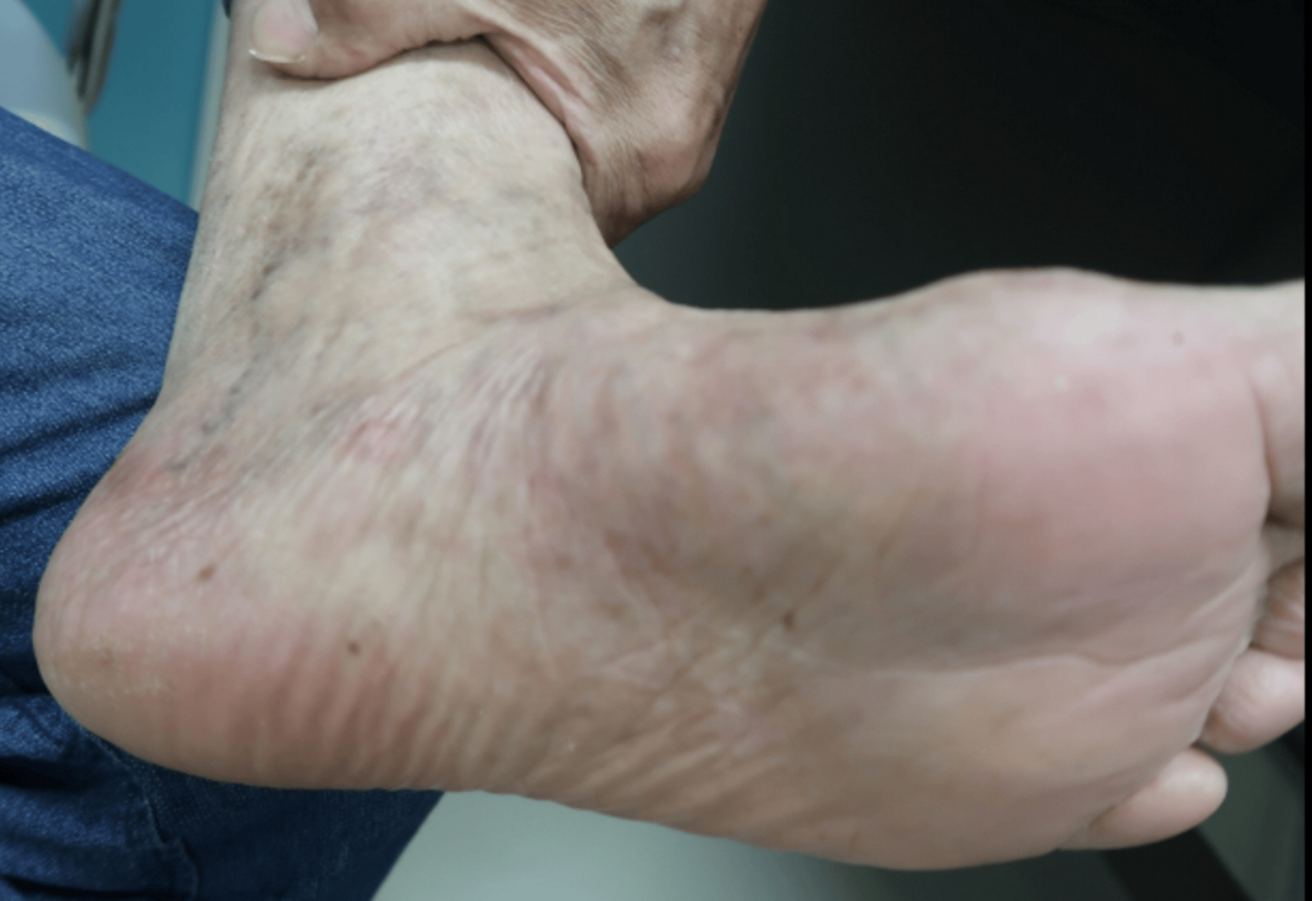
Seven weeks after resection of the lung cancer showing complete resolution of the bullous pemphigoid (BP) cutaneous lesions on the extremities

By seven weeks post-surgery, the cutaneous lesions had fully resolved, leaving only post inflammatory pigmentation (Figure 7).

## Discussion

While the relationship between BP and malignancy remains inconclusive despite several substantial studies, individual case reports offer intriguing insights. Baum et al. evaluated a cohort of 335 BP patients in comparison to the general population [4], and Aztmony et al. contributed with a systematic review and meta-analysis of this association [5]. He et al. attempted to establish causality through Mendelian randomization analysis using genome-wide association study (GWAS) data [6]. Nevertheless, none of these studies could definitively establish a causal link between BP and malignancy.

Ogawa et al. investigated the prevalence of internal malignancies among BP patients in Japan, finding an association rate of 5.8% for BP and 5.0% for pemphigus, both significantly elevated [7]. Among the 64 BP patients with associated malignancies, gastric cancer was the most frequently observed (14 cases), followed by lung cancer (seven cases) [7].

Furthermore, multiple case reports, especially involving BP patients with coexisting lung cancer, have shown that BP can partially or completely resolve following chemotherapy or tumor resection [8]. These findings suggest that treating the underlying malignancy may accelerate the resolution of BP lesions. For instance, carboplatin-based chemotherapy for metastatic SCC has been reported to improve BP symptoms within just three days [8].

Balestri et al. examined 40 BP cases, identifying seven with hematologic malignancies and 33 with solid tumors [9]. In 15 of these cases, BP onset preceded cancer diagnosis, consistent with our patient. Furthermore, 11 cases were resistant to standard BP treatments but responded following cancer management, emphasizing the need to consider paraneoplastic pemphigoid in BP cases that are refractory to conventional therapies.

To assist clinicians in identifying underlying malignancies in BP patients, Balestri et al. proposed clinical criteria, including early-onset BP, prior history of malignancy, cancer-related symptoms, and resistance to immunosuppressive therapies. These criteria provide a valuable framework for diagnosing and managing challenging BP cases [9].

The abnormal expression of the hemidesmosomal proteins BP230 and BP180 has been observed in various neoplastic conditions, suggesting that they play a role in tumor progression and invasion [10]. BP180 dysregulation has also been identified in several epithelial malignancies, including in SCC, as well as in cancers of the colon, pancreas, breast, and ovary. Importantly, increased BP180 expression in the stromal regions of non-small-cell lung carcinoma has been associated with greater metastatic potential [11].

In this case, the significant resolution of the BP lesions and the reduction in anti-BP180 antibody titers following lung cancer resection, without adjustments to the BP treatment, strongly support a paraneoplastic association. This highlights the importance of investigating malignancies in BP patients, particularly those unresponsive to standard therapies.

## Conclusions

This report provides further evidence of a potential link between BP and underlying malignancies - in this case, lung cancer. The incidental discovery of a right lung mass on routine chest imaging followed by the resolution of BP lesions and constant decrease in antibody titers after tumor resection without alterations to the BP treatment regimen strengthens the hypothesis that BP may act as a paraneoplastic syndrome in some cases.

Our findings emphasize the importance of thorough cancer screening in BP patients, particularly those with atypical presentations or a poor response to standard treatments. A comprehensive evaluation for malignancy should be considered as part of the diagnostic approach to BP, especially in treatment-resistant cases. The early detection of underlying cancer may significantly affect treatment decisions and patient outcomes. In conclusion, this case highlights the need for clinicians to maintain high suspicion for associated malignancies when managing BP, potentially leading to more timely interventions and improved patient care.
